# Supplementary figures and images for: The effects of Pilates vs. aerobic training on cardiorespiratory fitness, isokinetic muscular strength, body composition, and functional tasks outcomes for individuals who are overweight/obese: a clinical trial
Source: PeerJ. 2019 Feb 28;7:e6022. doi: 10.7717/peerj.6022 (PMC6397755; doi:10.7717/peerj.6022)

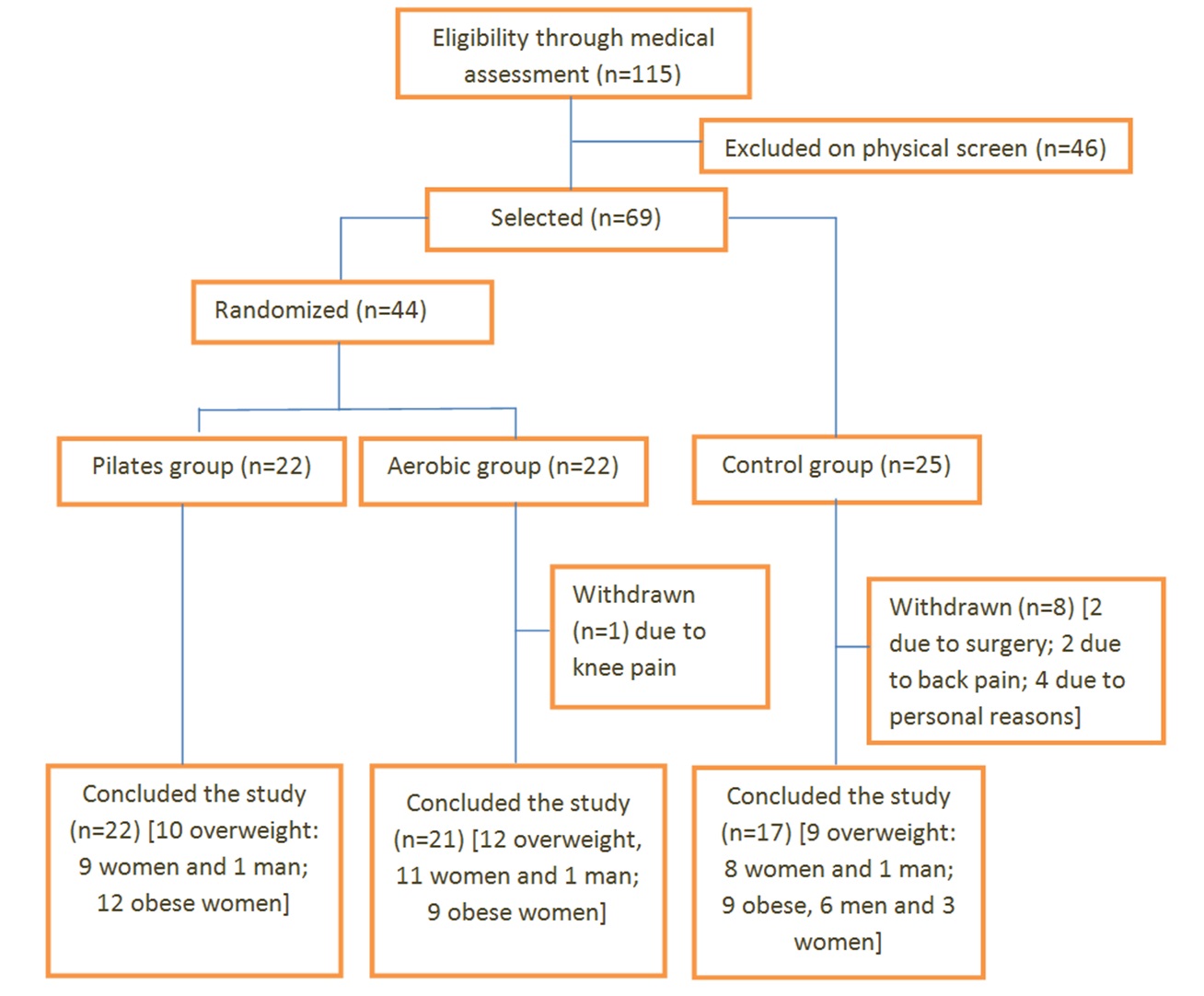

Supplement: Supplemental Information 2 [file peerj-07-6022-s002.jpg]
