# Supplementary material for: The effects of Pilates vs. aerobic training on cardiorespiratory fitness, isokinetic muscular strength, body composition, and functional tasks outcomes for individuals who are overweight/obese: a clinical trial
Source: PeerJ. 2019 Feb 28;7:e6022. doi: 10.7717/peerj.6022 (PMC6397755; doi:10.7717/peerj.6022)
Supplement: Supplemental Information 4 [file peerj-07-6022-s004.pdf]

# Pilates vs. aerobic training for individuals who are overweight/obese Version 2

**Angeles Rayes,Claudio Andre Barbosa de Lira,Ricardo Borges Viana,Rodrigo Luiz Vancini,Naryana Mascarin,Marilia Santos Andrade**

## Abstract

**Objective:** To compare the effects of *Pilates* and aerobic training on cardiorespiratory fitness, isokinetic muscular strength, body composition, and functional tasks outcomes for individuals who are overweight/obese.

**Design:** A prospective study.

**Settings:** University laboratory and *Pilates* Studio.

**Participants:** Sixty participants were divided into three groups: *Pilates* (n=22), Aerobic (n=21) and Control (n=17) groups.

**Interventions:** The *Pilates* and Aerobic groups attended 60-min exercise sessions, three times per week for 8 weeks. The Aerobic group performed walking training at heart rate corresponding to the ventilatory threshold. Pilates groups performed exercises on the floor, on the resistance apparatus, and 1-kg Dumbbells. The control group received no intervention. All three groups were evaluated before and after 8 weeks of training.

**Results:** There was no significant difference pre and post intervention in calorie intake [F(2, 57)=0.02744, p=0.97)]. A significant improvement of oxygen uptake at ventilatory threshold (p=0.001; d=0.60), respiratory compensation point (p=0.01; d=0.48), and maximum effort (p=0.01; d=0.33) was observed only in the *Pilates* group. Isokinetic peak torque for knees flexor and extensor muscles did not change for any groups. Lean mass (p=0.0005; *d*=0.19) and fat mass (p=0.0001; *d*=0.19) improved only in the *Pilates* group. Circumference measurements (waist and hip) decreased similarly in both experimental groups. Abdominal test performance improved more in the *Pilates* group (p=0.0001; *d*=1.69) than in the Aerobic group (p=0.03; *d*=0.95). Trunk extensor endurance and flexibility improved only in the *Pilates* group (p=0.0003; *d*=0.80 and p=0.0001; d=0.41, respectively). The *Pilates* group showed greater improvement on the chair and stair tests (p=0.0001; *d*=1.48 and p=0.002; *d*=0.78, respectively) than the Aerobic group (p=0.005; *d*=0.75 and p=0.04; *d*=0.41, respectively).

**Conclusion:** *Pilates* training can be used as an alternative physical training method for individuals who are overweight/obese since it promotes significant effects in cardiorespiratory fitness, body composition, and functional tests performance.

**Citation:** Angeles Rayes,Claudio Andre Barbosa de Lira,Ricardo Borges Viana,Rodrigo Luiz Vancini,Naryana Mascarin,Marilia Santos Andrade Pilates vs. aerobic training for individuals who are overweight/obese. **protocols.io**
dx.doi.org/10.17504/protocols.io.p5wdq7e
**Published:** 15 May 2018



This is an open access protocol distributed under the terms of the Creative Commons Attribution License, which permits unrestricted use, distribution, and reproduction in any medium, provided the original author and source are credited

# Protocol

Inclusion criteria:

**Step 1.**

- Body mass index > 25 kg/$m^2$;
- at least six months without practicing any kind of physical activity

Exclusion criteria:

**Step 2.**

- Present neurological, cognitive, orthopedic, respiratory, and/or endocrine disease (except for obesity).

Randomization:

**Step 3.**

Participants were randomly assigned by drawing their names written in pieces of paper from an opaque envelope and placing them in one of two experimental groups (*Pilates* or Aerobic groups).

Flowchart

**Step 4.**



This is an open access protocol distributed under the terms of the Creative Commons Attribution License, which permits unrestricted use, distribution, and reproduction in any medium, provided the original author and source are credited

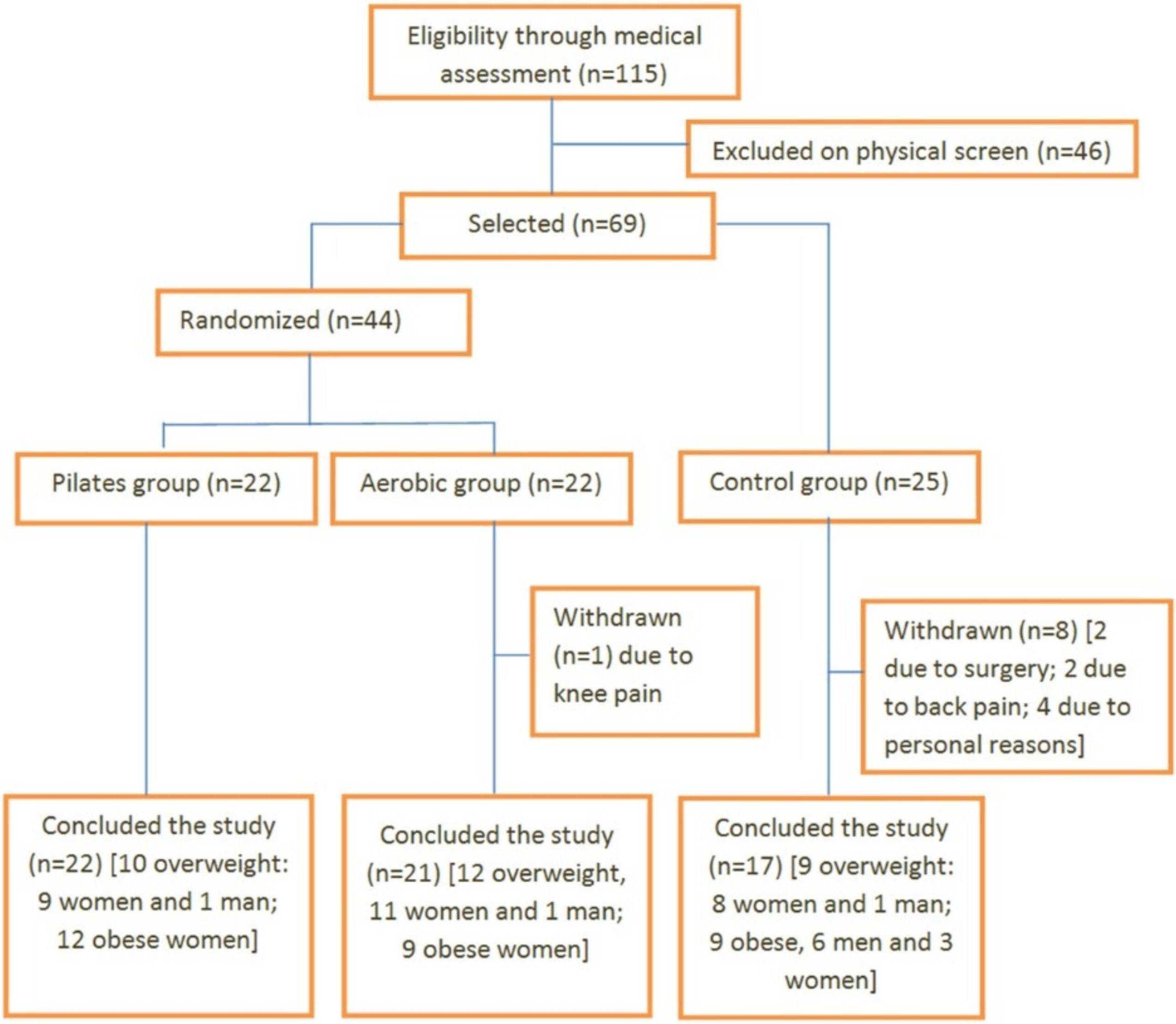


## Study design

**Step 5.**

- The study was carried out by blinded professionals involved with the tests and measurements.
- Due to the nature of the interventions, it was not possible to blind the participants and coaches involved in the training period. However, the professionals involved with the tests and measurements were blinded and made aware of allocation details only after data analysis.
- Participants in the Aerobic and the *Pilates* groups followed an experimental protocol for a period of eight weeks, with 60 minutes sessions three times per week (totaling 24 sessions) and were asked to not initiate any other exercise activity apart from the physical activity proposed by the study.
- When a participant missed three or fewer training sessions, the sessions were replaced at the end of the period, but when more than three sessions were missed, the participant was excluded from the study.

## Assessments:

**Step 6.**

All participants from the three groups underwent tests performed over two consecutive days:



This is an open access protocol distributed under the terms of the Creative Commons Attribution License, which permits unrestricted use, distribution, and reproduction in any medium, provided the original author and source are credited

- Food intake assessment;
- Cardiorespiratory maximal treadmill test;
- Isokinetic strength testing;
- Body composition and anthropometry assessment;
- Abdominal endurance test;
- Trunk extensor endurance test;
- Flexibility test;
- Functional tests (stair and chair tests).

Food intake assement:

**Step 7.**

- Participants were asked to record their food intake for three days (two week days and one weekend day);
- The participants had to write down a description of the foods consumed, quantity per unit or portion sizes, type of preparation, and time of day;
- The quantitative assessment of the food record was carried out by calculating total energy consumption;
- Intake analysis was performed using the Diet Win Professional 2.0 software (Brazil) by an experienced nutritionist;
- Participants were instructed to avoid changing their diet habits during the experimental protocol.

Cardiorespiratory maximal treadmill test:

**Step 8.**

- Motorized treadmill (Inbrasport, ATL, Porto Alegre, Brazil);

- Ramp protocol:
  - One minute standing on the treadmill;
  - 3 minutes of warm-up at 3 km/h and 0 degree of inclination;
  - From the 4th minute of testing, both speed (0.5km/h) and inclination (0.5%) increased every 20 seconds;
  - At the 6th minute of the test, the participant reached the final speed (6km/h), while the inclination was increased every 20 seconds until the volunteer's exhaustion.
  - The same individual protocol for each volunteer was used in the retest.

- Individual subjective level of exertion was obtained using the Borg scale;

- VT, RCP, and ($VO_2$max/peak), ventilation and expired gases were measured breath by breath using a metabolic analyzer (Quark, Cosmed, Italy), and all the measured data was considered as a mean of 20 seconds for analysis;.

Internal training load:

**Step 9.**

- After each training session, Aerobic and *Pilates* groups were presented to a rating of perceived



This is an open access protocol distributed under the terms of the Creative Commons Attribution License, which permits unrestricted use, distribution, and reproduction in any medium, provided the original author and source are credited

exertion (RPE - Borg scale) adapted by Foster et al. (2001), to calculate the load of each session;

- Participants were asked about the rate of the overall intensity of the training session thirty minutes after the end of the activity;
- The result was multiplied by the duration of the session; thus, it produced an index to monitor the internal training load.
- Foster C, Florhaug JA, Franklin J, Gottschall L, Hrovatin LA, Parker S, et al. A new approach to monitoring exercise training. *J Strength Cond Res*. 2001;15(1):109-115.

Isokinetic Strength Testing:

**Step 10.**

- Before the isokinetic testing, the participants performed a 5-min warm-up on a cycle ergometer (Cybex Inc., Ronkonkoma, NY, USA) at a resistance level of 25 Watts, followed by low intensity dynamic stretching exercises for the hamstrings and quadriceps;
- Following the warm-up period, participants were placed on the isokinetic dynamometer (Biodex Medical Systems Inc., Shirley, NY, USA) to evaluate the isokinetic concentric strength of both lower limbs in a random order;
- Peak torque (PT) of knees flexor and extensor muscles (both dominant and non-dominant) in concentric activity was measured;
- Concentric activity was evaluated at 60º/s (the lowest speed in order to avoid high joint pressure while producing the highest torque values) and 240º/s (an angular speed closer to knee angular speed during the gait) separated by a one-minute rest;
- Low angular speed (60º/s) was tested first. Participants completed 3 submaximal trials before each test angular speed to familiarize themselves with the equipment, and 5 maximal repetitions to test at 60°/s and at 240°/s.

Body composition and anthropometry assessment:

**Step 11.**

- Body composition was assessed by dual-energy X-ray absorptiometry (DXA, software version 12.3, Lunar DPX, Madison, WI, USA) in order to measure fat free mass and fat mass;
- BMI was calculated by dividing body mass by height squared (kg/m$^2$);
- Two body circumferences were considered: waist circumference (in cm) was measured around the midpoint between the lower margin of the last palpable rib and the top of the iliac crest; hip measurement was taken at the maximum circumference over the buttocks.

Abdominal endurance test:

**Step 12.**

- Abdominal endurance was evaluated using the partial trunk flexion test:
  - The test was performed with the participant lying down with knees flexed at 90 degrees, feet flat on the floor and hands clasped behind the head;
  - The trunk was raised to the point where the shoulders were lifted from the mat;
  - The maximum number of repetitions in one minute was measured.

Trunk extensor endurance test:

**Step 13.**

- Subjects underwent the Sorensen test to evaluate isometric endurance of the trunk extensor muscles:



This is an open access protocol distributed under the terms of the Creative Commons Attribution License, which permits unrestricted use, distribution, and reproduction in any medium, provided the original author and source are credited

- The participant lay prone on an examination table with the upper border of the iliac crests aligned with the edges of the table;
- The lower body was secured by three belts located around the hip, knees and ankles;
- The arms were crossed over the chest;
- Participants were asked to isometrically maintain the upper body in a horizontal position as long as possible;
- The time to exhaustion was measured in seconds.

## Flexibility test:

**Step 14.**

- Flexibility was evaluated using the sit and reach test which specifically measures the flexibility of the lower back and hamstring muscles:
  - The participants sits on the floor with lower limbs fully extended, ankles flexed and feet flat against the box used for the test;
  - The participant must lean slowly and protrude forward as far as possible, sliding their fingers along a ruler affixed to the upper side of the box;
  - After two practice reaches, the participant reaches out and holds the position for two seconds while the distance (in centimeters) was recorded.

## Functional tests:

**Step 15.**

- The stair test (adapted from the American College of Sports Medicine (2013):
  - The subject must climb up and down a set of 12 steps of 16 cm in height (total of 24 steps) twice.
- Chair test (adapted from the American College of Sports Medicine (2013):
  - The subject must get up out of and sit back down into a 50 cm-high chair 10 times;
  - The runtimes was recorded in seconds.

American College of Sports Medicine. ACSM´s guidelines for exercise testing and prescription. 9th ed. Baltimore: Lippincott Williams & Wilkins; 2013.

## Intervention: Pilates Training Protocol

**Step 16.**

Table 1. List of Pilates exercises and apparatus

| **Program on Pilates** [1] | | |
|---|---|---|
| Name of Exercises | Number of repetitions | Evolution in weeks |
| Hundred | 1 | 4th – 8th |
| Single Leg Circles, Single Leg Stretch, Double Leg Stretch, Spine Stretch, Saw, Side Kick | 3– 5 | 1st – 8th |
| Single Leg Kick, Double Leg Kick, Spine Twist | 4 - 6 | 2nd – 8th |
| Single Straight Leg Stretch, Criss-Cross | 5 – 10 | 4th – 8th |
| Shoulder Bridge, Leg Pull Front | 3 – 6 | 4th – 8th |
| **Program on Reformer**[1] | | |
| Footwork, Tendon Stretch, Pelvic Lift, Running, | 5 – 10 | 1st – 8th |



This is an open access protocol distributed under the terms of the Creative Commons Attribution License, which permits unrestricted use, distribution, and reproduction in any medium, provided the original author and source are credited

| | | |
|---|---|---|
| Short Box Series: Round, Flat Back, Twist | 3 – 5 | 4th – 8th |
| Long Box Series: Pulling Straps, T | 5 – 8 | 4th – 8th |
| Knee Stretches Series: Round, Arched back | 5 – 8 | 4th – 8th |
| Long Stretches Series: Front, Elephant, Downstretch | 5 - 8 | 4th – 8th |
| Chest Expansion Kneeling, Reverse Chest Expansion, Splits Side, Stomach Massage: Flat back, Reach, Twist | 5 – 8 | 4th – 8th |
| **Program on Cadillac**[1] | | |
| Rolldown Bar, Breathing, Chest Expansion, Thigh Stretch, | 3 – 5 | 1st – 8th |
| Push Through Seated Front, Spread Eagle | 3 – 5 | 1st – 8th |
| Arms springs series (supine), Legs springs series (supine) | 3 -5 | 1st – 8 th |
| Side Lying series | 5 - 10 | 4th – 8th |
| Standing on Floor: Punching, Salute, Hug- a- tree, Butterfly | 3 – 6 | 4th – 8th |
| **Program on Chair**[1] | | |
| Double Leg Pumps, Single Leg Pumps, Standing Leg Pump Front – Side - Crossover | 5 – 10 | 1st – 8th |
| Washer Woman, Swan Front, Seated Mermaid | 3 - 5 | 4th – 8th |
| Frog Lying Flat, Single leg pump – Lying flat, Achilles stretch | 5 – 10 | 1st – 8th |
| **Program on Ladder Barrel**[2] | | |
| Horse Back | 3 | 4th – 8th |
| Short Box Series: Round, Flat Back and Twist | 3 - 5 | 4th – 8th |
| **Program with Dumbbells – 1kg**[2] | | |
| Wall – Circles, Sliding, Rolling down | 3 – 5 | 1st – 8th |
| Zip Up, Chest expansion, Shaving the head, Arm Circles, Biceps Curls (I, II), Triceps extension, The Bug, Boxing, Lunges | 5 – 10 | 2nd - 8th |
| **Program with Magic Circle**[1] | | |
| Arm Work, Leg Work and Head Work Series | 5 – 10 | 1st - 8th |

1 – List of Pilates Exercises & Equipment. Debora Lenssen. The PMA Pilates Certification Exam (Study Guide). 2 – Brooke Siler, Cristy Turlington. The Pilates Body: The Ultimate At-Home Guide to Strengthening, Lengthening and Toning Your Body - Without Machines. 2000. ISBN-13:9780767903967

## Intervention: Aerobic Training Protocol

**Step 17.**

- An individual training program was developed for each subject based on the HR corresponding to ventilatory threshold (VT), assessed in the cardiorespiratory maximal treadmill test;
- This program consisted of performing eight weeks of moderate aerobic training in a park with flat terrain three times a week, with sessions lasting for 60 minutes;
- Each one-hour session was divided into:
  - Warm up (10 minutes);
  - Walking in VT intensity (40 minutes) and;
  - Cool-down (10 minutes).
- All participants used a HR monitor (Suunto, Ambit, Finland) for monitoring HR during all training sessions, and the sessions were supervised all the time by a coach to ensure the HR had been maintained in the target value.



This is an open access protocol distributed under the terms of the Creative Commons Attribution License, which permits unrestricted use, distribution, and reproduction in any medium, provided the original author and source are credited
